# Diet quality in middle-aged and older women with and without body weight dissatisfaction: results from a population-based national nutrition survey in Switzerland

**DOI:** 10.1017/jns.2021.32

**Published:** 2021-05-25

**Authors:** Angéline Chatelan, Isabelle Carrard

**Affiliations:** Department of Nutrition and Dietetics, School of Health Sciences (HEdS-GE), University of Applied Sciences and Arts Western Switzerland (HES-SO), Rue des Caroubiers 25, 1227 Carouge-Geneva, Switzerland

**Keywords:** Body weight dissatisfaction, Diet quality, Dietary intakes, Dieting, Healthy eating index, Middle-aged women, National nutrition surveys, Older women

## Abstract

Body weight dissatisfaction is associated with unhealthy dietary behaviours in young adults, but data are scarce regarding how this relationship evolves with age. The objectives of the present study were to assess the prevalence of body weight dissatisfaction and the association between body weight dissatisfaction, nutrient intake and diet quality in middle-aged and older women. We used data of a population-based sample of 468 middle-aged (50–64 y/o) and older (65–75 y/o) women, extracted from the cross-sectional 2014–15 Swiss National Nutrition Survey. Body weight dissatisfaction was assessed by questionnaire. Dietitians assessed dietary intakes using two non-consecutive computer-assisted multi-pass 24-h dietary recalls and performed anthropometric measurements. Nutrient intakes were calculated and compared with national dietary guidelines, and diet quality scored with the 2010 Alternate Healthy Eating Index (2010-AHEI). 41⋅1 % of women reported body weight dissatisfaction, and 49⋅8 % wanted to lose weight. Body weight dissatisfaction was associated with weight loss desire and a higher body mass index (BMI; *P* < 0⋅001). Women with body weight dissatisfaction consumed significantly less carbohydrates and dietary fibres, even when BMI was controlled for (*P* < 0⋅05). They also fell short of national dietary guidelines for magnesium and iron. Body weight dissatisfied women obtained lower 2010-AHEI scores than satisfied women (*β* −4⋅36, 95 % CI −6⋅78, −1⋅93). However, this association disappeared when the BMI was introduced in the equation. This highlights the importance of targeting both body dissatisfaction and unhealthy eating in obesity prevention and treatment at all ages.

## Introduction

Body dissatisfaction, which comprises dissatisfaction or concern with weight and shape, has been associated with a wide range of psychological disorders and unhealthy behaviours^(1)^. Particularly in female adolescents and young women, body dissatisfaction is a predictor of depression and of disordered eating, including extreme dieting behaviours, unhealthy weight control behaviours and binge eating^(2)^. The frequent association of body dissatisfaction with unhealthy weight control behaviours might prevent those who desire to lose weight to adequately manage their weight^(3,4)^. Because body dissatisfaction is a major risk factor for eating disorders^(5)^, studies on its correlates have mainly included female adolescents or young adults. Whereas one could think that body image and its correlates is an issue specific to the period of adolescence, longitudinal studies highlighted that body dissatisfaction and unhealthy weight control behaviours stay stable when transiting from adolescence to adulthood^(6)^.

Recent studies documented that weight-control diets and fear of weight gain were observable across the entire adult lifespan^(7,8)^. The stigmatisation that surrounds overweight and obesity in Western societies and pressure to conform to thin beauty ideals can explain why most adult women who perceive themselves as overweight are also body dissatisfied^(9)^. While controlling for body mass index (BMI), the stability of body dissatisfaction was observed in middle-aged and older women until the age of 75. In a large survey in the US including 5868 women of 25–89 y/o, 89 % in the 45–54 y/o group, 89 % in the 55–64 y/o group and 88 % in the 65–74 y/o group reported body dissatisfaction^(10)^. In a sub-study including over 1800 women aged 50 and more, 79 % of them reported a moderate to important role of weight and shape concerns in their self-perception, and over 70 % reported dissatisfaction with current weight and shape^(11)^. Moreover, 71 % of these women were trying to lose weight and 36 % spent half of their time or more dieting in the last past 5 years. The presence of more concerning behaviours in these women, such as taking diet pills, extreme dieting behaviours, following low-calorie diets or skipping meals, suggest that maladaptive behaviours associated with the drive for thinness observed in adolescents may not systematically decrease with age^(12)^.

These potentially unhealthy weight control behaviours are of concern particularly when it comes to ageing women. Weight management is recommended at all ages, but extreme dieting behaviours reinforce specific risks already present with older age, such as nutritional deficiencies (folate, calcium, zinc and vitamins D, B1, B6 and B12) or sarcopenia^(13,14)^. Ageing can be very heterogeneous between individuals, and it is particularly influenced by lifestyle factors^(15)^. For example, nutrient deficiencies are associated with frailty^(16)^, while healthy dietary patterns prevent from cardiovascular diseases^(17,18)^ and prospectively predicts longevity^(18–20)^.

To summarise, the prevalence of body dissatisfaction might be associated with unhealthy weight management practices, also in middle-aged and older women. But studies are scarce^(21)^ and concern with weight and shape has been mainly assessed in convenience samples in women of this age. Yet, high percentages of them still adopt behaviours in order to lose weight, revealing a dissatisfaction with their body weight. Moreover, the association of body weight dissatisfaction with nutrient intakes and diet quality in middle-aged or older women is not known, while such an association would be important particularly at this age. The objectives of the present study were to assess the prevalence of body weight dissatisfaction, willingness to lose weight and dieting, the characteristics of women with body weight dissatisfaction, as well as the association between body weight dissatisfaction and nutrient intake, adherence to dietary guidelines and diet quality, in a population-based sample of women aged 50–75 y/o, with data issued from a national nutrition survey intended to be representative of the Swiss population.

## Methods

### Population and study design

Data were extracted from the first Swiss National Nutrition Survey (menuCH), a cross-sectional study that comprises a population-based sample of 2086 adults aged 18–75 y/o recruited between January 2014 and February 2015^(22,23)^. menuCH primarily explores dietary intakes and weight status in the general population living in Switzerland and includes people with a sufficient command of German, French or Italian (i.e. the three main national languages). A written invitation was sent home to a representative sample of the Swiss population from the national sampling frame for personal and household surveys^(24)^. People who consented to participate in the study had a face-to-face interview in one of the ten study centres and a phone interview 2–6 weeks later with a registered dietitian specifically trained for the study. In addition, participants completed a paper questionnaire. In total, 469 women aged 50–75 y/o were recruited. For our analyses, one woman was excluded because of a missing questionnaire (0⋅2 %), yielding a final sample of 468 women. More information about the survey menuCH is available here: https://menuch.iumsp.ch. The present study was conducted according to the guidelines laid down in the Declaration of Helsinki and all procedures involving research study participants were approved by the research commission of the Canton de Vaud (lead committee in Lausanne, Switzerland, Protocol 26/13, approved on 12 February 2013). Written informed consent was obtained from all participants.

### Dietary intake assessment

menuCH assessed dietary intake by two computer-assisted multi-pass 24-h dietary recalls (24HDR) using the validated program GloboDiet® (formerly EPIC-Soft®)^(25,26)^. Seven women had only one 24HDR. Recalls were non-consecutive and spread over all weekdays and seasons. To support survey participants in quantifying food intake, dietitians used a book with 119 series of six graduated portion-size pictures^(27)^ and a set of about 60 actual household measures. According to the description, each food item was linked to the best match in an extended research version of the 2015 Swiss Food Composition Database^(28)^, using the software FoodCASE (Premotec GmbH, Winterthur, Switzerland)^(29)^. Further information about dietary assessment methods and data quality controls (e.g. misreporting) has been published in previous articles^(22,23)^. The national dietary guidelines^(30)^, mostly adapted from the 2019 dietary guidelines for Germany, Austria and Switzerland^(31)^, were used to compare nutrient intakes with recommendations.

### Diet quality definition

To assess overall diet quality, we used the 2010 Alternate Healthy Eating Index (2010-AHEI), which is a composite score made up of eleven food and nutrient components (Supplementary Table S1 of Supplementary material)^(20)^. Each component scored between 0 (worst) and 10 (optimal diet quality). The total score was up to 110 if (1) intakes of vegetables, fruit, whole grains (defined as a carbohydrate-to-fibre ratio ≤10:1), nuts and legumes, fish (proxy for long-chain *n*-3 fatty acids) and polyunsaturated fatty acids were high; (2) intakes of sweetened beverages and fruit juices, red and processed meats, *trans*-fat and sodium were low and (3) intake of alcoholic drinks was moderate (see cut-offs in Supplementary Table S1)^(20,32)^. This index has been used extensively in the literature to study the association between diet quality and several chronic diseases, as well as cause-specific and all-cause mortality^(20,33)^.

### Body weight satisfaction

In the questionnaire, participants selected one of four possible answers (very satisfied – satisfied – dissatisfied – very dissatisfied) in response to the question about body weight satisfaction (Are you currently satisfied with your body weight?). For further analyses, participants were classified as being either ‘satisfied’ or ‘dissatisfied’ with their body weight. Participants were also asked about their weight desire: ‘Which statement defines you best?’ (possible answers: ‘I would like to lose weight’, ‘I would like to maintain my current weight’ or ‘I would like to gain weight’). Because of the low rate of responses ‘I would like to gain weight’, this category was grouped with ‘I would like to maintain my current weight’ for further analyses, resulting in two categories ‘weight loss desire – yes’’ and ‘weight loss desire – no’. Finally, their current and past dieting practices were assessed as follows: ‘Are you currently on a diet to lose weight?’ and ‘In the last 12 months, have you been on a weight-loss diet?’ (possible answers: ‘Yes’ or ‘No’).

### Anthropometry and obesity markers

Body weight, height, and waist and hip circumferences were measured following an international protocol^(34)^. Briefly, body weight and height were measured to the nearest 0⋅1 kg and cm without shoes and heavy clothes, using a calibrated Seca 701 scale equipped with a Seca 220 telescopic measuring rod (Seca GmbH, Hamburg, Germany). For waist and hip circumferences, we calculated the mean of three consecutive measurements taken to the nearest 0⋅1 cm using a Gulick I unstretchable tape (North Coast Medical, CA, USA). Abdominal obesity was defined as having a waist-to-hip ratio equal to or above 0⋅85^(35)^.

### Demographics and other variables

Survey participants reported their birthdate, nationality, education (highest degree) and self-reported health in the questionnaire. They were also asked how many times a day they took snacks (solid foods) between main meals during a standard week (Monday to Sunday). Finally, participants reported on which days they usually skipped breakfast in a standard week (Monday to Sunday). In the analyses, participants were considered as skippers if they skipped breakfast at least 4 d in a standard week (i.e. more than half of the weekdays).

### Statistical analyses

Food and nutrient intakes were estimated using 2-d means. Descriptive results were presented using medians [P25–P75] for continuous variables due to evidence of non-normality (Shapiro–Wilk tests for BMI, the number of snacks and intake of 15 nutrients; *P* < 0⋅001). All seventeen histograms showed positively skewed distributions, confirming the need for using non-parametric tests. Categorical variables were presented as percentages. For bivariate analyses, differences in ordinal and continuous variables were assessed using Mann–Whitney *U* tests and in categorical variables using *χ*^2^ tests or Fisher's exact tests, when one or more expected cell counts in the cross-tabulation were less than 5. We used multiple quantile regressions to assess differences in nutrient intakes between women with and without body weight satisfaction, respectively, multiple logistic regressions for differences in adherence to national dietary guidelines. To assess the association between diet quality using the 2010-AHEI (normally distributed) and body weight dissatisfaction, multiple linear regressions were computed. Between-group differences were considered statistically significant at *P* < 0⋅05. All statistical analyses were carried out using STATA version 14 (Stata Corp, College Station, TX, USA).

## Results

### Prevalence of body weight dissatisfaction, willingness to lose weight and dieting

In all, 41⋅1 % of middle-aged and older women in the Swiss population, divided between 45⋅2 % of middle-aged (50–64 y/o) and 33⋅9 % of older women (65–75 y/o), were very dissatisfied or dissatisfied with their weight (Table 1) and 49⋅8 % wanted to lose weight. Comparisons of both age group categories showed that older women aged 65–75 y/o were less dissatisfied with their body weight than middle-aged women aged 50–64 y (*P* = 0⋅005). The percentage of women currently dieting was low (6⋅0 %) and not different between the two groups, but a lower percentage of older women (*P* 0⋅030) reported having been on a weight-loss diet during the last 12 months (6⋅4 %) in comparison with middle-aged women (12⋅8 %).

**Table 1.** Prevalence in women aged 50–75 years, Swiss National Nutrition Survey 2014–15

	Women aged 50–75 years	Women aged 50–64 years	Women aged 65–75 years	*P*-value^a^
*N*	468	297	171	
Body weight satisfaction				
Very dissatisfied	10⋅3 %	12⋅5 %	6⋅4 %	**0·005**
Dissatisfied	30⋅8 %	32⋅7 %	27⋅5 %	
Satisfied	36⋅1 %	35⋅0 %	38⋅0 %	
Very satisfied	22⋅9 %	19⋅9 %	28⋅1 %	
Weight desire				
Willingness to lose weight	49⋅8 %	55⋅6 %	39⋅8 %	**0·002**
Willingness to maintain weight	49⋅6 %	43⋅4 %	60⋅2 %	
Willingness to gain weight	0⋅6 %	1⋅0 %	0⋅0 %	
Currently dieting				
On a diet at survey time	6⋅0 %	7⋅1 %	4⋅1 %	0·191
No dieting at survey time	94⋅0 %	92⋅9 %	95⋅9 %	
Dieting in the past year				
On a diet in the last 12 months	10⋅5 %	12⋅8 %	6⋅4 %	**0·030**
No dieting in the last 12 months	89⋅5 %	87⋅2 %	93⋅6 %	

Values in bold indicate statistically significant results.

a Differences between middle-aged and older women assessed by Mann–Whitney *U* test (ordinal variables), *χ*^2^ test, respectively, Fisher's exact test (binary variables).

### Characteristics of women with body weight dissatisfaction

The percentages of middle-aged (96⋅3 %) and older (89⋅7 %) women who wanted to lose weight were higher among women with body weight dissatisfaction than among those who were satisfied with their weight (*P* < 0⋅001; Table 2). Dissatisfied women were also more likely to be on a weight-loss diet currently or in the last year in both age groups (*P* ≤ 0⋅001). The BMI of women with body weight dissatisfaction was significantly higher compared with women with body weight satisfaction: 67⋅2 and 82⋅8 % of middle-aged and older women with body weight dissatisfaction, respectively, suffered from overweight or obesity (*P* < 0⋅001). Concordant differences were observed for abdominal obesity, with higher percentages of women with body weight dissatisfaction having a waist-to-hip ratio higher than 0⋅85 (*P* < 0⋅001). Of note, 31⋅3 % of middle-aged women and 17⋅2 % of older women with body weight dissatisfaction had a weight in the normal range. Regarding eating behaviours, the number of snacks per day or skipping breakfast was not different between women who were satisfied or dissatisfied with their body weight in either age group. Self-reported health was rated lower in women with body weight dissatisfaction in both age groups (50–64 years: *P* < 0⋅001; 65–75 years: *P* = 0⋅009). Finally, middle-aged women with lower education were more prone to body weight dissatisfaction (*P* = 0⋅048). Because characteristics of women with body weight dissatisfaction were similar among 50–64 y/o and 65–75 y/o (except for education), both age groups were further analysed together.

**Table 2.** Characteristics of middle-aged and older women with and without body weight dissatisfaction

	Women aged 50–64 years			Women aged 65–75 years		
	Dissatisfied	Satisfied	*P*-value^1^	Dissatisfied	Satisfied	*P*-value^a^
*N*	134	163		58	113	
Weight loss desire (%)						
Yes	96⋅3 %	22⋅1 %	**<0·001**	89·7 %	14·2 %	**<0·001**
No	3·7 %	77·9 %		10·3 %	85·8 %	
Dieting (%)						
Yes (currently or in the last year)	21·6 %	7·4 %	**<0·001**	17·2 %	2·7 %	**0·001**
No	78·4 %	92·6 %		82·8 %	97·3 %	
Body Mass Index (BMI, kg/m^2^)						
Median [P25–P75]	27·2 [24·0–30·7]	21·8 [20·5–23·4]	**<0·001**	27·7 [25·8–30·8]	23·0 [21·0–24·7]	**<0·001**
BMI categories (%)						
Underweight (BMI <20 kg/m^2^)	1·5 %	17·8 %	**<0·001**	0·0 %	13·3 %	**<0·001**
Normal (20 ≤ BMI < 25 kg/m^2^)	31·3 %	67·5 %		17·2 %	63·7 %	
Overweight (25 ≤ BMI < 30 kg/m^2^)	35·1 %	13·5 %		46·6 %	20·4 %	
Obese (BMI ≥ 30 kg/m^2^)	32·1 %	1·2 %		36·2 %	2·7 %	
Abdominal obesity (%)						
Waist-to-hip ratio ≥ 0·85	38·8 %	6·7 %	**<0·001**	47·4 %	15·0 %	**<0·001**
Waist-to-hip ratio < 0·85	61·2 %	93·3 %		52·6 %	85·0 %	
Snacking (number of snacks/day)						
Median [P25–P75]	2·0 [1·0–2·6]	1·4 [1·0–2·0]	0·109	1·0 [0·3–2·0]	1·0 [1·0–2·0]	0·385
Skipping breakfast ≥ 4 out of 7 days (%)						
Yes	14·2 %	11·0 %	0·415	6·9 %	1·8 %	0·085
No	85·8 %	89·0 %		93·1 %	98·2 %	
Self-reporting health status (%)						
Bad and very bad	1·5 %	0·0 %	**<0·001**	1·7 %	1·8 %	**0·009**
Medium	23·9 %	4·9 %		20·7 %	14·2 %	
Good	50·7 %	55·2 %		67·2 %	53·1 %	
Very good	23·9 %	39·9 %		10·3 %	31·0 %	
Nationality (%)						
Swiss	92·5 %	91·4 %	0·723	96·6 %	97·3 %	0·771
Foreigner	7·5 %	8·6 %		3·4 %	2·7 %	
Education: Highest degree (%)						
Only mandatory school or no degree	9·7 %	3·1 %	**0·048**	3·4 %	6·2 %	0·586
Secondary (e.g. apprenticeship)	58·2 %	58·9 %		70·7 %	63·7 %	
Tertiary (e.g. university)	32·1 %	38·0 %		25·9 %	30·1 %	

Values in bold indicate statistically significant results.

a Differences between dissatisfied and satisfied women assessed by *χ*^2^ test, respectively, Fisher's exact test (categorical variables), Mann–Whitney *U* test (ordinal and continuous variables).

### Body weight dissatisfaction and nutrient intake

Regarding macronutrient intake (Table 3), women aged 50–75 y/o with body weight dissatisfaction had lower median intakes of carbohydrates (*P* = 0⋅050), sugars (*P* = 0⋅005) and dietary fibres (*P* < 0⋅001). Regarding micronutrient intake, no differences emerged among women with or without body weight dissatisfaction, except a lower folate intake in women with body weight dissatisfaction (*P* = 0⋅029). After adjustment for age, BMI, self-reported health status and education (model 1), only carbohydrates (*P* = 0⋅022) and dietary fibres (*P* = 0⋅009) were significantly different. The additional control for total energy intake in the equation (model 2) equalised the difference of carbohydrate intakes between groups.

**Table 3.** Daily nutrient intakes of women aged 50–75 years with and without body weight dissatisfaction

	Crude^a^			Model 1^a^			Model 2^a^		
	Dissatisfied (*N* 192)	Satisfied (*N* 276)	*P*-value	Dissatisfied (*N* 192)	Satisfied (*N* 276)	*P*-value	Dissatisfied (*N* 192)	Satisfied (*N* 276)	*P*-value
	Median [P25–P75]	Median [P25–P75]		Adjusted median	Adjusted median		Adjusted median	Adjusted median	
Energy (kcal)	1771 [1497–2061]	1832 [1530–2171]	0·292	1764	1823	0·370	–	–	–
Proteins (g)	67·4 [54·5–81·4]	64·8 [55·3–79·5]	0·238	68·3	64·9	0·702	69·0	66·3	0·216
Carbohydrates (g)	173·5 [138·7–209·1]	188·4 [147·8–236·2]	**0·050**	173·5	188·1	**0·022**	179·2	197·3	0·407
Sugars (g)	80·0 [58·1–107·8]	92·6 [68·2–118·2]	**0·005**	79·1	91·7	0·053	80·9	95·9	0·149
Total fat (g)	75·1 [56·3–90·3]	76·6 [60·6–93·6]	0·585	74·5	76·3	0·872	75·6	78·5	0·284
Saturated fat (g)	28·8 [20·3–37·9]	28·4 [22·3–35·7]	0·762	28·5	28·9	0·809	28·9	29·6	0·754
Monounsaturated fat (g)	18·2 [13·2–24·6]	19·9 [13·3–26·6]	0·157	18·0	19·5	0·482	18·8	19·9	0·970
Polyunsaturated fat (g)	6·2 [4·4–8·9]	6·9 [5·0–10·3]	0·053	6·4	7·0	0·440	6·7	7·2	0·783
Dietary fibres (g)	16·8 [13·0–21·5]	19·7 [15·8–25·6]	**<0·001**	16·9	20·1	**0·009**	17·4	20·7	**0·012**
Calcium (mg)	595·2 [423·0–829·2]	608·1 [448·5–784·6]	0·780	586·7	611·8	0·239	604·1	610·1	0·801
Potassium (mg)	1982 [1557–2390]	2109 [1686–2589]	0·120	1996	2129	0·239	2009·5	2139·4	0·316
Sodium (mg)	2251 [1725–2920]	2342 [1777–2962]	0·386	2296	2367	0·069	2307·6	2346·0	0·789
Magnesium (mg)	190·8 [150·1–227·1]	199·3 [161·3–252·1]	0·263	191·3	198·2	0·417	191·9	203·7	0·540
Iron (mg)	6·2 [4·6–7·6]	6·5 [5·2–8·3]	0·293	6·2	6·5	0·358	6·3	6·7	0·998
Folate (μg)	193·8 [149·6–255·6]	216·9 [162·2–285·7]	**0·029**	200·0	217·1	0·392	198·5	217·5	0·433

Values in bold indicate statistically significant results.

a Adjusted medians and differences between dissatisfied and satisfied women estimated with quantile regressions without adjustment (Crude), with adjustment for age, body mass index, self-reported health status, education (model 1) and total energy intake (model 2).

### Body weight dissatisfaction and adherence to dietary guidelines

**Fig. 1.**
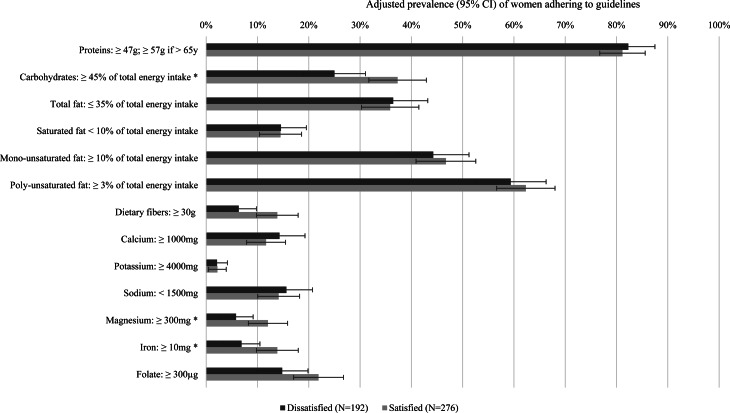
Adherence to national dietary guidelines in women aged 50–75 years with and without body weight dissatisfaction (*P-value < 0⋅05, adjusted prevalences (95 % CI) and differences between dissatisfied and satisfied women estimated with logistic regressions adjusted for age, body mass index, self-reported health status and education).

Macro- and micronutrient intakes in body weight dissatisfied and satisfied women were compared with national dietary guidelines (Fig. 1). In general, the percentages of women following dietary recommendations were under 50 % for most items except protein and polyunsaturated fat intakes. Moreover, lower percentages of women with body weight dissatisfaction reached the guidelines for carbohydrates, magnesium and iron compared with women with body weight satisfaction, after adjustment for age, BMI, self-reported health status and education (*P* < 0⋅05).

### Associations between body weight dissatisfaction and diet quality index

Regression analyses showed a bivariate relationship between body weight dissatisfaction and diet quality, assessed with the 2010-AHEI (Table 4). Women with body weight dissatisfaction obtained a lower diet quality score than women with body weight satisfaction (*β* −4⋅36; 95 % CI −6⋅78, −1⋅93). Supplementary Table S1 of Supplementary material shows that women with body weight dissatisfaction ate less fruit, less nuts and legumes, more red and processed meat, and had less moderate intake of alcoholic drinks (*P* < 0⋅05). They also tended to consume less whole-grain products (*P* = 0⋅06) and less sugar-sweetened beverages and fruit juices (*P* = 0⋅14; Table 4). Age and total energy intake (model 1) did not modify the relationship between body weight dissatisfaction and the diet quality score. When BMI was introduced in the regression model (model 2), the relationship between body weight dissatisfaction and diet quality did not reach significance anymore. In model 3 including body weight dissatisfaction, age, total energy intake, BMI, education and self-reported health status, BMI was the only significant predictor of diet quality (*β* −0⋅44 per 1 kg/m^2^ increase; 95 % CI −0⋅75, −0⋅12). This highlights that body weight dissatisfaction was associated with poorer diet quality, mainly through higher BMI.

**Table 4.** Associations between diet quality (2010-AHEI^a^) and body weight dissatisfaction in women aged 50–75 years

	Crude^b^	Model 1^b^	Model 2^b^	Model 3^b^
	*β* (95 % CI)	*β* (95 % CI)	*β* (95 % CI)	*β* (95 % CI)
*Body weight satisfaction*	*0 (ref)*	*0 (ref)*	*0 (ref)*	*0 (ref)*
Dissatisfaction	−4·36* **(-6·78, −1·93)**	−4·52* **(−6·97, −2·08)**	−1·73 (−4–71, 1·24)	−1·66 (−4·68, 1·35)
Age (per 10 years increase)		0·31 (−1·29, 1·91)	0·54 (−1·05, 2·13)	0·58 (−1·04, 2·19)
Total energy intake (per 100 kcal/d increase)		−0·21 (−0·46, 0·05)	−0·21 (−0·47, 0·05)	−0·22 (−0·48, 0·04)
Body mass index (kg/m^2^)			−0·49* **(−0·80, −0·19)**	−0·44* (**−0·75, −0·12)**
*Self-reported health status: Bad and very bad*				*0 (ref)*
Medium				−1·31 (−13·23, 10·62)
Good				1·32 (−10·36, 13·01)
Very good				0·43 (−11·41, 12·27)
*Education: Secondary (apprenticeship)*				*0 (ref)*
Mandatory school				−4·68 (−9·90, 0·53)
Tertiary (university)				0·02 (−2·57, 2·61)
Constant	51·52* (50·00, 53·08)	53·45* (42·11, 64·80)	63·25* (50·49, 76·02)	61·44 (42·95, 79·93)

Values in bold indicate statistically significant results.

a Modified Alternate Healthy Eating Index 2010.

^b^Differences between dissatisfied and satisfied women were assessed using multiple linear regressions (*P < 0·05).

## Discussion

The present study revealed that 41 % of Swiss middle-aged and older women were dissatisfied with their body weight and that 50 % of the sample wanted to lose weight. Women with body dissatisfaction consumed less carbohydrates, and dietary fibres, even when controlling for age, BMI, self-reported health status and education as covariates. Except for protein and polyunsaturated fat, guidelines were hard to follow for a high percentage of middle-aged and older women, with lower adherence regarding carbohydrates, magnesium and iron among women with body weight dissatisfaction. Diet quality, assessed by the 2010-AHEI, showed a bivariate association with body weight dissatisfaction, but this association was mainly explained by higher BMI in people with body weight dissatisfaction.

### Prevalence of body weight dissatisfaction

The percentages of body weight dissatisfaction found in a Swiss population-based sample (50–64 y/o: 45 % and 65–75 y/o: 34 %) were lower than those reported among a convenience sample of American women in ages categories 45–74, which were close to 90 %^(10)^. In the national cohort study Nutrinet-Santé, which may be culturally closer to the present sample as France is a neighbouring country of Switzerland, the percentage of French adult women (ages 18 and more) declaring being body dissatisfied was 51 %^(36)^. Together with culture, the sampling methods and the questionnaire wording may have played a role in the differences found between the USA, France and Switzerland. These however high percentages of body dissatisfied women are concerning, considering that body dissatisfaction and weight concerns in adult women are associated with an increased risk of eating disorders and other unhealthy weight management behaviours^(4,37)^, together with poorer mental health (e.g. lower self-esteem or depression)^(38–40)^. In the present study, body weight dissatisfaction and desire to lose weight were slightly lower in older women than in middle-aged women. These results are in line with the reduction of weight concerns with age observed in previous studies^(41)^, even though the percentages of women with overweight increased slightly with age.

### Characteristics of women with body weight dissatisfaction

Body weight dissatisfaction was highly associated with higher BMI and a desire to lose weight in both age groups. Yet, a much smaller percentage of these dissatisfied women declared being currently dieting or having undertaken a weight-loss diet during the previous year. These findings are consistent with the paradoxical effect, showing that overweight perception and desire to lose weight does not help improve weight management behaviours^(42)^. Weight stigma seems to increase the motivation to lose weight and simultaneously decrease the perceived self-efficacy to do so, which may explain the discrepancy between intention and action undertaken to lose weight^(43)^.

### Body weight dissatisfaction and nutrient intake

The present results showed that women with body weight dissatisfaction had lower carbohydrates and fibres intakes than those with body weight satisfaction, even when controlling for covariates such as age or BMI. Dissatisfied women aged from 50 to 75 seem more inclined to watch starchy and sugary foods than fatty foods to manage their weight (in gram and percentage of total energy intake). Of note, 15 % of women with body weight dissatisfaction had a diet poor in carbohydrates (<30 % of total energy intake) against 9 % among those who were satisfied (*P* = 0⋅07, data not shown). This might be a persistence of the weight-loss low-carb diets commonly used in the 70s–80s. A second explanation for the lower carbohydrate intake in women with body weight dissatisfaction could be their habits of restraining dietary intake on some days (e.g. recorded 24HDR days) and compensating on other days. Rigid dietary control has indeed been associated with binge-eating and disinhibited eating^(44)^. This may explain why a larger percentage of women with body weight dissatisfaction, who had slightly lower energy intakes on recorded days, suffered from overweight or obesity. Of note, binge-eating and disinhibition are difficult to capture with traditional dietary assessment methods, such as the 24HDR, first because they occur occasionally, and second, because they are associated with shame^(45)^.

The micronutrient intakes of middle-aged and older women with body weight satisfaction were closer to the dietary guidelines than those of women with dissatisfaction, particularly regarding magnesium and iron. This is of concern because nutritional adequacy has a role to play in healthy ageing and in preventing the progression of chronic diseases and undernutrition with age^(14,19,46)^.

### Body weight dissatisfaction and overall diet quality

Greater body weight dissatisfaction was associated with a reduced 2010-AHEI score of about 4 points, whatever the age or the total energy intake. The use of a diet quality index shed light on the general imbalance of the overall diet quality in women with body weight dissatisfaction. This association was, however, mainly explained by higher BMI, which was related to both body weight dissatisfaction and lower diet quality. In spite of the desire to lose weight associated with a higher BMI, middle-aged and older women reduced carbohydrates but did adopt unhealthier dietary behaviours (e.g. less fruit, less nuts and legumes, more red and processed meat), suggesting that body weight dissatisfaction may be as distressing for middle-aged and older women as it is for the younger population.

### Clinical and public health implications

The present findings highlight that body weight dissatisfaction is related to unhealthy dietary intakes in older age, similarly to adolescence and young adulthood^(4,37)^. This also raises the question of how to promote healthy weight management in older age groups. Because accurate weight perception seems to decrease with age, it has been suggested that healthcare providers should help older adults with excess weight recognise their weight status, in order to encourage them to manage their weight^(47)^. The present results indicate that this should be done with tact to avoid triggering body weight dissatisfaction, and the same counterintuitive results on weight loss that have been observed in adolescents and young adults^(3)^. This is especially important knowing that a relatively large proportion of middle-aged (31 %) and older (17 %) women with body weight dissatisfaction in the sample were in the normal-weight category. This is concerning because restrictive diets without medical need may lead to severe consequences, such as eliminating important dietary sources of vitamins and minerals or disordered eating^(48)^.

### Strengths and limitations

The present study had some limitations. The data collection was cross-sectional, preventing any causal interpretation. The participation rate was low: of 5496 eligible people reachable by phone, only 38 % responded^(22)^, limiting the external validity of the study. The questions regarding body weight satisfaction, weight loss desire and dieting practices were limited in the questionnaire; however, similar questions have been used in large survey with relevant findings^(40)^. The question ‘In the last 12 months, have you been on a weight-loss diet?’ could have induced memory bias among older participants. The selection of a sub-sample of women aged 50–75 reduced the sample size for the present study. Nutrient intake from food supplements was not considered in our analyses as not assessed in menuCH. Furthermore, self-reported nutrient intakes should be correlated to objective biomarkers to better assess the risk of nutrient deficiencies (not available in menuCH). As for strengths, these data came from a national survey with a recruitment strategy based on stratified random population-based sampling. Dietary intake assessment was carried out with two computer-assisted multi-pass 24HDR, using the validated program GloboDiet®^(25,26)^, all these elements speaking in favour of the good internal validity of the present study. Finally, adopting two different strategies to analyse dietary intakes, i.e. nutrient intakes and diet quality index, turned out to show complementary results.

## Conclusions

The present study highlighted that body weight dissatisfaction affects about 40 % of women aged 50–75 y/o. Women with body weight dissatisfaction had lower carbohydrate intake and healthy dietary intakes than those with satisfaction. Body weight dissatisfaction was highly correlated with BMI but also observable in a relatively large proportion of women with normal weight. Higher BMI was the main predictor of poor diet quality, pointing out the overlapping of body weight dissatisfaction and excess weight, which is a characteristic of the Western society. These observations raise the question of how to promote healthy dietary behaviours at all ages of life and how to encourage the feeling of self-efficacy necessary to initiate behaviour change. Healthcare providers and public health practitioners should be careful when approaching the topic of weight loss to avoid fueling body weight dissatisfaction, but rather target health, even among middle-aged and older women. The results of the present study reinforce the call from scholars to integrate prevention messages issued from the eating disorder field that target both body dissatisfaction and unhealthy dieting within the field of obesity prevention and treatment^(49)^.

## Supplementary material

For supplementary material accompanying this paper visit http://dx.doi.org/10.1017/jns.2021.32. click here to view supplementary material
