# Supplementary material for: Diet quality in middle-aged and older women with and without body weight dissatisfaction: results from a population-based national nutrition survey in Switzerland
Source: J Nutr Sci. 2021 May 25;10:e38. doi: 10.1017/jns.2021.32 (PMC8342191; doi:10.1017/jns.2021.32)
Supplement: Supplementary file 1 [file S204867902100032Xsup001.docx]

| **Supplementary Table S1:** Components and scoring method of the modified 2010 Alternate Healthy Eating Index^1,2^ and mean scores (±SD) in middle-aged and older women, Swiss National Nutrition Survey, 2014-2015. | | | | | | | | | | | | | | | | | | | | | |
| --- | --- | --- | --- | --- | --- | --- | --- | --- | --- | --- | --- | --- | --- | --- | --- | --- | --- | --- | --- | --- | --- |
| **Components** | **Included food items** | **Criteria for minimum score (0)** | **Criteria for maximum score (10)** | **Women**  **aged 50 to 75 years** | | | | | | | | |  | | | | | | | | |
|  |  |  |  | **Dissatisfied**  **(N=192)** | | | | **Satisfied**  **(N=276)** | | | | |  | | | |  | | | | |
|  |  |  |  | **Mean** ± | | **SD** |  | **Mean** ± | | **SD** |  | **P-Value^11^** |  | |  |  |  | |  |  |  |
| Vegetables (sevings/day)^3^ | All vegetables, leafy vegetables, sprouts, green beans, peas, sweat corn, root vegetables, cabbages, avocadoes, mushrooms, onions, seaweeds, homemade vegetable soups  Except: potatoes products, olives, herbs, vegetables juices | 0 | ≥ 5 | 3.5 | ± | 2.1 |  | 3.6 | ± | 2.0 |  | 0.523 |  |  |  |  |  |  |  |  |  |
| Fruit (servings/day)^4^ | All fruits  Except: fruit juices, fruit jams, candied fruit | 0 | ≥ 4 | 4.1 | ± | 3.1 |  | 4.9 | ± | 3.1 |  | **0.006** |  |  |  |  |  |  |  |  |  |
| Whole grains (g/day) | All bread products, flours, breakfast cereals, cereal flakes and brans, dough, pasta, rice, spätzle, other cereal grains (e.g., quinoa, barley) with a carbohydrate-to-fiber ratio smaller than 10:1 | 0 | ≥ 75 | 3.4 | ± | 3.6 |  | 4.1 | ± | 3.7 |  | 0.060 |  |  |  |  |  |  |  |  |  |
| Sugar-sweetened beverage & fruit juice (servings/day)^5^ | Sweetened soft drinks, sports and energy drinks, fizzy drinks, diluted syrup, ice tea, alcoholic drinks substitutes, drinks made with fruit juices (e.g.  lemonades, nectars), 100% fruit juices, smoothies  Except: drinks with artificial sweeteners (e.g. light or sugar-free soft drinks) | ≥ 1 | 0 | 6.8 | ± | 4.1 |  | 6.3 | ± | 4.2 |  | 0.142 |  |  |  |  |  |  |  |  |  |
| Nuts & legumes (servings/day)^6^ | Nuts, seeds, legumes, meat substitutes, soy products | 0 | ≥ 1 | 1.9 | ± | 3.3 |  | 2.8 | ± | 3.7 |  | **0.006** |  |  |  |  |  |  |  |  |  |
| Red & processed meat (servings/day)^7^ | Fresh meat of mammals, offal, wild meat, sausages, cold cuts, smoked and cured meat | ≥ 1.5 | 0 | 4.3 | ± | 3.8 |  | 5.6 | ± | 3.8 |  | **<0.001** |  |  |  |  |  |  |  |  |  |
| Trans fatty acids (% of total energy intake)^8^ | Not applicable | ≥ 4 | ≤ 0.5 | 9.4 | ± | 0.4 |  | 9.4 | ± | 0.4 |  | 0.563 |  |  |  |  |  |  |  |  |  |
| Fish, excluding processed products (g/day) | Fish, seafood  Except: processed fish (e.g., fish in crumbs), seafood products (e.g., surimi) | 0 | 32.4 | 2.6 | ± | 4.1 |  | 2.9 | ± | 4.4 |  | 0.342 |  |  |  |  |  |  |  |  |  |
| Polyunsaturated fatty acids (% of total energy intake) | Not applicable | ≤ 2 | ≥ 10 | 2.1 | ± | 1.9 |  | 2.3 | ± | 2.0 |  | 0.315 |  |  |  |  |  |  |  |  |  |
| Sodium (mg/day)^9^ | Not applicable | highest decile | lowest decile | 5.1 | ± | 3.2 |  | 5.0 | ± | 3.2 |  | 0.637 |  |  |  |  |  |  |  |  |  |
| Alcohol (drinks/day)^10^ | Beer, wine, champagne, wine products, port, sherry, vermouth, cocktails, liquors, spirits, long drinks | ≥ 2.5 | 0.5 - 1.5 | 4.0 | ± | 3.5 |  | 4.7 | ± | 3.7 |  | **0.033** |  |  |  |  |  |  |  |  |  |
| **Total** | Not applicable | **0** | **110** | 47.2 | **±** | 13.8 |  | 51.5 | **±** | 12.6 |  | **<0.001** |  |  |  |  |  |  |  |  |  |
| ^1^ Adapted from Chiuve et al. 2012 and Pestoni et al. 2018 | | |  |  |  |  |  |  |  |  |  |  |  |  |  |  |  |  |  |  |  |
| ^2^ Intermediate food intake was scored proportionately between the minimum score 0 and the maximum score 10. | | | | | | | | | | | | | |  |  |  |  |  |  |  |  |
| ^3^ One serving was equal to 118.3g of raw or cooked vegetables, 30g of dried vegetables or 250g of homemade vegetable soup. | | | | | | | | | | | | | | | | | | |  |  |  |
| ^4^ One serving was equal to 118.3g of raw or cooked fruit or 30g of dried fruit. | | | | | | |  |  |  |  |  |  |  |  |  |  |  |  |  |  |  |
| ^5^ One serving was equal to 226.8g. | |  |  |  |  |  |  |  |  |  |  |  |  |  |  |  |  |  |  |  |  |
| ^6^ One serving was equal to 28.4g. | |  |  |  |  |  |  |  |  |  |  |  |  |  |  |  |  |  |  |  |  |
| ^7^ One serving was equal to 113.4g of red meat or 42.5g of processed meat. | | | | |  |  |  |  |  |  |  |  |  |  |  |  |  |  |  |  |  |
| ^8^ According to the Swiss regulation, each food item must contain max. 2g of trans fat per 100g of total fat | | | | | | | | | | | | |  |  |  |  |  |  |  |  |  |
| ^9^ Values in highest and lowest decile were ≥ 3763 and ≥ 3503 mg/d and ≤ 1381 and ≤ 1274 mg/d in women aged 50 to 64 years, respectively in women aged 65 to 75 years | | | | | | | | | | | | | | | | | | | | | |
| ^10^ One drink was equal to 113.4g of wine, 340.2g of beer or 42.5g of liquor/spirit. A score of 2.5 was given to non-drinkers. | | | | | | | | | | | | | | | | | |  |  |  |  |
| ^11^ Differences between dissatisfied and satisfied women assessed by independent t-tests. | | | | | | |  |  |  |  |  |  |  |  |  |  |  |  |  |  |  |
